# Characterization of Infarct Size and Remodeling Using CMR and PET in Mice Models of Reperfused and Non-Reperfused Myocardial Infarction

**DOI:** 10.3390/diagnostics15232960

**Published:** 2025-11-22

**Authors:** Jose Gavara, Tamara Molina-Garcia, Mustafa Ezzeddin, Ana Diaz, Nerea Perez-Sole, Maria Ortega, Victor Marcos-Garces, Elena de Dios, Antoni Bayes-Genis, Amparo Ruiz-Sauri, Cesar Rios-Navarro, Vicente Bodi

**Affiliations:** 1 Centro de Investigación Biomédica en Red–Cardiovascular, 28029 Madrid, Spain; jose.gavara@outlook.es (J.G.); ana.diaz@uv.es (A.D.); neere_8@hotmail.com (N.P.-S.); marcos_vic@gva.es (V.M.-G.); elenaddll@gmail.com (E.d.D.); antonibayesgenis@gmail.com (A.B.-G.); ruizsa@uv.es (A.R.-S.); 2 INCLIVA Biomedical Research Institute, 46010 Valencia, Spain; orcarma@alumni.uv.es; 3 Heart Institute, Germans Trias i Pujol University Hospital, 08916 Badalona, Spain; 4 Medicine Department, Faculty of Medicine, University of Valencia, 46010 Valencia, Spain; tamara5mg1999@gmail.com; 5 Central Biomedical Research Unit, University of Valencia, 46010 Valencia, Spain; mustafa.ezz@uv.es; 6 Cardiology Department, Hospital Clínico Universitario, 46010 Valencia, Spain; 7 Medicine Department, Autonomous University of Barcelona, 08193 Barcelona, Spain; 8 Pathology Department, Faculty of Medicine, University of Valencia, 46010 Valencia, Spain

**Keywords:** magnetic resonance, myocardial infarction, positron emission tomography

## Abstract

**Background/Objectives**: Unlike post-mortem histopathology, cardiovascular magnetic resonance (CMR) and positron emission tomography (PET) enable longitudinal assessment of structural, functional, and metabolic alterations in preclinical myocardial infarction models. This study aims to describe the temporal evolution of infarct size and systolic function by CMR and glucose consumption via PET, explore their differences in non-reperfused and reperfusion infarction models, and assess their capacity to predict histology-derived infarct size and systolic function at chronic phase CMR. **Methods**: Two murine models of myocardial infarction were generated using permanent (non-reperfused, *n* = 8) or transient (reperfused, *n* = 9) coronary occlusion. CMR and fluorine-18 2-fluoro-2-deoxyglucose PET imaging were performed at baseline and at 1, 7, and 21 days post-infarction to quantify infarct size, systolic function, and myocardial glucose metabolism. Infarct size was also assessed using Masson’s trichrome staining. **Results**: At 24 h post-infarction, CMR-derived infarction together with significant reduction in systolic function and glucose metabolism were already noted in both models. At 21-day CMR, however, reperfused mice showed lower infarct size and more preserved systolic function compared to their non-reperfused counterparts, while no differences in glucose metabolism were reported. Infarct size and systolic function at 1-day CMR and the number of segments with reduced glucose consumption at 1-day PET independently predicted histology-derived infarct size and long-term systolic function. **Conclusions**: Combined PET/CMR imaging enables non-invasive, sequential evaluation of infarct size, systolic function, and glucose metabolism in experimental myocardial infarction. This multimodality approach is well suited for assessing the efficacy of emerging therapies in preclinical research.

## 1. Introduction

Myocardial infarction (MI) arises from acute coronary artery occlusion, most commonly due to thrombus formation following plaque rupture, and results in irreversible myocardial injury caused by sustained ischemia and hypoxia [1,2]. Although mortality rates have improved, survivors remain at elevated risk for recurrent MI, heart failure, and arrhythmias [3,4], highlighting the need for adjunctive strategies to prevent maladaptive post-infarction remodeling. Experimental animal models are indispensable for elucidating molecular mechanisms and evaluating emerging therapeutic interventions [5,6,7]; however, successful clinical translation has been limited [6,7]. Progress in this field depends on the availability of precise, reproducible methods to characterize structural and functional changes after MI.

Histological analysis offers detailed post-mortem assessment but precludes longitudinal evaluation. In contrast, non-invasive imaging modalities, particularly cardiovascular magnetic resonance (CMR) [8,9,10,11,12,13] and positron emission tomography (PET) [12,14,15,16], enable serial evaluation of myocardial function, viability, perfusion, and metabolism, providing critical insights into the dynamic repair process. Despite their prior application in preclinical research [8,9,10,12,17], temporal changes in CMR- and PET-derived parameters across different MI models remain insufficiently defined.

Given the importance of exploring new therapeutic approaches beyond timely coronary reperfusion, a more comprehensive understanding of these non-invasive imaging techniques is crucial. We therefore performed sequential CMR and 18F-2-fluoro-2-deoxyglucose PET in two mouse MI models, transient and permanent coronary ligation. Our aims were two-fold: First, to describe the evolution of infarct size and systolic function quantified by CMR and glucose consumption determined via 18F-2-fluoro-2-deoxyglucose PET and compare them between the two models; Second, to scrutinize the predictive capacity of infarct size and systolic function on 1-day CMR and the number of segments with reduced glucose consumption on 1-day PET as regards histology-derived infarction size and long-term systolic function.

## 2. Materials and Methods

Animal protocols were performed following European Parliament Directive 2010/63/EU guidelines and were approved by the institutional review board (Protocol number: 2022/VSC/PEA/0259). C57BL/6J mice (Mus musculus) [aged 16 ± 2 weeks] were supplied by Charles River Laboratories (Chatillon-sur-Chalaronne, France). All animals were acclimatized to the experimental facility for at least three weeks before surgery. During this period, the mice were bred and maintained under specific pathogen-free conditions at a constant temperature of 22 ± 2 °C and humidity of 60–65% with a 12 h dark/light cycle, and with free access to normal chow and autoclaved water. General health status, feeding behaviors, and activity were monitored daily by trained researchers to ensure physiological stability and the absence of distress or illness. Only animals exhibiting normal behavior and stable body weight were included in the experimental protocol.

### 2.1. Mouse MI Model

Mice (*n* = 26; 1:1 sex ratio) were randomly allocated to one of the following experimental groups: (i) sham; (ii) non-reperfused MI; (iii) reperfused MI. Randomization was stratified by sex and performed using a computer-generated sequence. Allocation was conducted by personnel not involved in procedures or analysis to ensure blinding. All investigators responsible for surgery, imaging, and data analysis were blinded to group assignment until study completion.

MI was induced by ligation of the left anterior descending coronary artery and two MI models were performed: (a) non-reperfused MI (permanent coronary ligation without reperfusion, *n* = 8) and (b) reperfused MI (transient 45 min coronary artery occlusion followed by reperfusion, *n* = 9). To monitor the animals during the procedure, an electrocardiogram was recorded prior to surgery and after ischemia induction using an Indus MouseMonitor S system surgical monitor (Indus Instruments, Webster, TX, USA). The sham group (*n* = 5) underwent the same surgical protocol except for coronary artery occlusion. Four animals died within the first day after surgery.

As per protocol, the knot was tightened to occlude the coronary artery. In the reperfused MI model, a 23G tube was placed between the coronary artery and the 6-0 silk suture and was later removed to allow complete reperfusion following the ischemic period. The tube was positioned through the sixth intercostal space to the exterior of the animal. In both models, the thorax was closed once ischemia onset was confirmed both visually and by electrocardiogram. In the reperfused MI model the tube was removed after 45 min of occlusion to allow coronary reperfusion confirmed on electrocardiogram. The duration of ischemia was set at 45 min, as recommended by current guidelines for experimental models of myocardial ischemia. Shorter occlusion times produce minimal changes in myocardial structure and function, whereas occlusions longer than two hours often cause near-complete infarction of the area at risk [6,7].

Intraperitoneal buprenorphine (0.05 mg/kg, twice daily) and meloxicam (0.3 mg/kg, once daily) were administrated for five days after surgery. Animal well-being was monitored daily. Further details of the experimental protocol can be consulted elsewhere [18,19].

### 2.2. CMR/18F-2-Fluoro-2-deoxyglucose PET Protocol

CMR and 18F-2-fluoro-2-deoxyglucose PET images were sequentially acquired at baseline (prior to surgery) and at days 1, 7, and 21 after the experimental procedure in all three (one sham and two MI) experimental groups.

Simultaneous CMR/18F-2-fluoro-2-deoxyglucose PET imaging was performed on a fully integrated system (MR Solutions, Guildford, UK) consisting of a cryogen-free, dry magnet 3.0-T MRI scanner (MRS*DRYMAG 3017-3.0T) with gradient strength (2000 mT/m) combined with a PET scanner (MRS*PET CLIP-ON).

The animals were anesthetized with isoflurane anesthesia (2% in 100% oxygen; Abbott Laboratories, Chicago, IL, USA) during the entire protocol. A volume of 0.1 mmol/kg of gadolinium-diethylenetriaminepentaacetic acid (Magnograf, Juste S.A.Q.F., Madrid, Spain) and 0.2–0.3 mCi in 0.2 mL of 18F-2-fluoro-2-deoxyglucose (Advanced Accelerator Applications Molecular Imaging Iberica, Murcia, Spain) was added to 0.2 mL of saline and intraperitoneally injected [20] using body weight-adjusted contrast volume and administered dose (2-fluoro-2-deoxyglucose activity). Intraperitoneal injection of contrast was selected in line with a previous study demonstrating that, unlike in humans, MI mice submitted to CMR showed similar myocardial T1 values regardless of administration route [20].

Afterwards, mice were placed in a supine position on a pre-heated (37 °C) stage. CMR/PET imaging time was 60 ± 4 min and no complications were recorded.

PET and CMR images were reconstructed in 2D with the VivoQuant^TM^ 2022 software (version 5.3.0, inviCRO, Needham, MA, USA).

### 2.3. CMR Acquisition and Quantification

Cine CMR imaging acquisitions were first performed. A low-resolution gradient echo scout scan with zero offset was used to determine the position of the heart in the scanner in coronal, axial, and sagittal directions and afterwards cine images were acquired. A multi-slice short-axis flash sequence (field of view 40 × 40 mm^2^, matrix 256, slice thickness 1 mm, pulse repetition time/echo time 16/0 msec, flip angle 25°) was used to acquire 4–5 slices in a mid-left ventricle (LV) position, perpendicular to the long axis of the heart. The same sequence was used to acquire 2- and 3-chamber slices. Six temporal frames were acquired per cardiac cycle.

Late gadolinium enhancement (LGE) imaging was performed in the same projections used for the cine images, 45 min after contrast injection, employing a multi-slice short-axis flash sequence (field of view 40 × 40 mm^2^, matrix 256, slice thickness 1 mm, pulse repetition time/echo time 16/0 msec, flip angle 25°). The Retrospective triggering graphical user interface software (Gustav Strijkers, Amsterdam UMC, version 9.1.1, Amsterdam, the Netherlands) was used for retrospectively gated scanning. The six temporal frames of each mid-LV slice were visually reviewed to properly identify tele-systolic and tele-diastolic frames for subsequent reconstruction study.

CMR-derived LV volumes and mass were determined by manual planimetry of LV endocardium and epicardium on short-axis slices (CMR42, Circle Cardiovascular Imaging Inc., version 5.17.1, Calgary, AB, Canada) on end-diastolic and end-systolic frames to determine LV end-diastolic volume index, LV end-systolic volume index, LV ejection fraction and LV mass.

Infarct size (% of LV mass) was quantified by manual planimetry using LGE imaging (CMR42, Circle Cardiovascular Imaging Inc., Calgary, AB, Canada). Segments were classified as with LGE if the enhancement difference with remote segments in LGE images was higher than five standard deviations. The 16-segment model was employed on a segmental basis to calculate CMR-derived wall thickness, presence of hypokinesia, and LGE using CMR42 (Circle Cardiovascular Imaging Inc., Calgary, AB, Canada).

### 2.4. PET Acquisition and Quantification

Sixty minutes after 18F-2-fluoro-2-deoxyglucose injection, 15 min static PET images were acquired. PET reconstructions were performed for the complete 15 min scan. A 3D region of interest within LV tissue was manually delineated using PMOD version 2.95 software (PMOD Technologies LLC, Zurich PET Center, Zurich, Switzerland) and expressed as a standardized uptake value (SUV) obtained after using a correction factor (Activity [kBq]/Weight [g]). Abnormal activity concentration was defined qualitatively (normal vs. low) as regions or segments showing low activity concentration if the difference with remote segments was higher than 5 standard deviations. Low activity segments were confirmed in both short and long-axis views.

The two investigators performing cardiac function analyses were blinded to the protocol applied.

### 2.5. Histological Analysis

For histologic evaluation, mice were euthanized by cervical dislocation under isoflurane anesthesia, followed by heart isolation for cardiac assessment. Cardiac samples were stained with hematoxylin–eosin (Sigma Aldrich, St. Louis, MO, USA) for histological analysis and with Masson’s trichomic staining (PanReac AppliChem, Chicago, IL, USA) to detect fibrosis and quantify infarct size [18,19].

Briefly, hearts were removed after sacrifice and cut into 1 mm thick short-axis slices. Myocardial slices were fixed in 4% paraformaldehyde acid, embedded in paraffin, sectioned (5 μm) and mounted on double gelatin-coated glass slides. After staining with hematoxylin–eosin and Masson’s trichomic, samples were scanned using a Pannoramic 250 Flash III Scanner (3DHISTECH, Budapest, Hungary), and next all microscopic images were quantified manually offline in a dedicated laboratory by a trained observer blinded to the experimental protocol applied using Image ProPlus 7.0 software (Media Cybernetics Inc., Rockville, MD, USA).

### 2.6. Statistical Analysis

Data were tested for normal distribution using the Kolmogorov–Smirnov test. Continuous normally distributed data were expressed as mean ± standard deviation of the mean and compared using paired and unpaired Student’s *t*-tests or one-way ANOVA. Non-parametric data were expressed as the median with the interquartile range and compared using the Mann–Whitney U-test or Kluskal–Wallis. Group percentages were compared using Chi-square test or Fisher’s exact test where appropriate. All data points were included in the analysis, and no exclusions were made.

Spearman’s rank-order or Pearson correlation coefficients were used to assess associations between the main CMR, PET, and histological indices.

For overall analysis, we used the Youden index applied to receiver operating curve analyses to determine the best cut-off points for dichotomizing continuous variables to identify MI mice and depressed LV ejection fraction (LVEF) (<40%) at 21 days (CMR imaging at 1 day: LVEF < 40%, infarct size > 0% of LV mass and/or ≥1 segment, hypokinesia ≥ 1 segment; PET imaging: SUV < 1.44 × 10^−5^, low concentration of 18F-2-fluoro-2-deoxyglucose ≥ 1 segments).

Inter-observer variability in the measurement of CMR- and PET-derived indices was obtained by calculating the differences between the measurements performed by the two operators in 15 CMR randomly selected studies. Intra-observer variability in calculating CMR and PET indices was determined by comparing the differences between two repeated measurements carried out by the operator from the study group (with an interval of 1 month from the first to the second measurement) in 15 CMR studies. Inter- and intra-observer variability (absolute and relative changes and intraclass correlation coefficients) was calculated. Intraclass correlation coefficient values were interpreted according to conventional thresholds (poor < 0.5, moderate 0.5–0.75, good 0.75–0.9, excellent > 0.9).

Statistical significance was achieved at a two-tailed *p*-value < 0.05. The SPSS statistical package (version 15.0, SPSS Inc., Chicago, IL, USA) was used throughout.

We conducted an observational study aimed at providing a comprehensive assessment of myocardial structure and function. Given the descriptive nature of our approach, we did not specify a primary outcome measure.

## 3. Results

### 3.1. CMR Imaging

Clinical characteristics (sex, age, and weight) as well as baseline CMR and PET indices, were comparable between control animals and the baseline measurements of both the reperfused and non-reperfused MI groups (Table 1).

**Figure 1:**
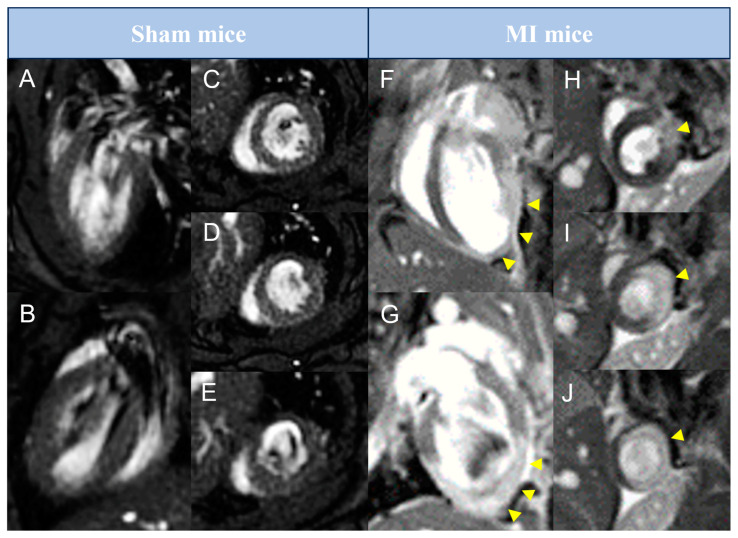
Representative pictures of late gadolinium enhancement–cardiovascular magnetic resonance studies performed in preclinical mice models. Animals from sham (**A**–**E**) or MI (**F**–**J**) groups were submitted to cardiovascular magnetic resonance imaging 21 days after surgery. Representative pictures in long-axis (**A**,**B**,**F**,**G**) and in basal to apical short-axis (**C**–**E**,**H**–**J**) are shown. Arrowheads represent areas with late gadolinium enhancement. MI = Myocardial infarction.

Mice in the two MI groups and sham controls underwent sequential CMR and PET imaging at baseline and at 1, 7, and 21 days after MI induction. Representative long-axis and short-axis CMR images from control, reperfused, and non-reperfused animals at day 21 are presented in Figure 1.

Inter- and intra-observer variability for the PET and CMR measurements used in this study are summarized in Table 2. Intraclass correlation coefficients exceeding 0.90 indicate excellent measurement reproducibility.

**Figure 2:**
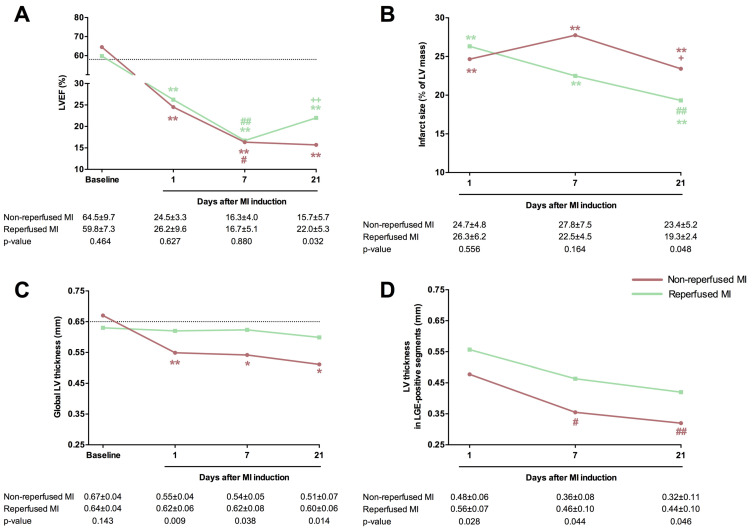
Comparison of the main CMR indices in mice submitted to permanent or transient coronary artery ligation. One control group (*n* = 5) and two mice MI models were formed by permanent coronary ligation (non-reperfused MI, *n* = 8) or transient 45 min (reperfused MI, *n* = 9) coronary occlusion. All animals (sham and both MI groups) underwent CMR studies at baseline and 1, 7, and 21 days after MI induction. LVEF (**A**), infarct size (**B**), global LV thickness (**C**), and LV thickness in LGE-positive segments (**D**) were determined. The dotted line represents the corresponding value in the control group. Data were expressed as mean ± standard deviation of the mean and compared using unpaired Student’s *t*-test for inter-group comparisons and one-way ANOVA for intra-group comparisons. * *p* < 0.05, ** *p* < 0.01 vs. baseline; # *p* < 0.05, ## *p* < 0.01 vs. 1 day; + *p* < 0.05, ++ *p* < 0.01, vs. 7 days. CMR = Cardiovascular magnetic resonance. LGE = Late gadolinium enhancement. LV = Left ventricle. LVEF = Left ventricular ejection fraction. MI = Myocardial infarction.

A substantial decline in systolic function occurred in both MI models one day after coronary occlusion, as evidenced by elevated LV end-diastolic volume index and LV end-systolic volume index, reduced LVEF, and a higher number of hypokinetic segments than the corresponding baseline values (Table 3, Figure 2). CMR tests conducted during sub-acute (7-day) and chronic (21-day) phases illustrated progressive LVEF decay and LV volume dilatation, with most LV systolic function decline occurring within the first week after the experimental procedure. Specifically, mice undergoing non-reperfused and reperfused MI experienced a drop in LVEF of 33% at the 1-day CMR test (*p*-value < 0.05) and 36% at one week (*p*-value < 0.01). Scrutinizing the two MI groups 21 days after the procedure, mice subjected to transient coronary ischemia exhibited more preserved LVEF (reperfused MI group: 22.0 ± 5.3%, non-reperfused MI group: 15.7 ± 5.7%, *p*-value = 0.032), yet LV end-diastolic and LV end-systolic volume indices showed no differences (Table 3, Figure 2). Conversely, the sham group exhibited no time-related changes in systolic function (Figure 2). In terms of myocardial structure, no LGE-positive segments were observed in baseline studies. Although infarct size at 1-day CMR was similar in both MI groups (reperfused: 26.3 ± 6.2% of LV mass; non-reperfused: 24.7 ± 4.8% of LV mass), mice experiencing permanent coronary ischemia showed greater infarct extent at the chronic (21-day) phase (reperfused MI: 19.3 ± 2.4% of LV mass; non-reperfused MI: 23.4 ± 5.2% of LV mass, *p*-value = 0.048). Regarding LV thickness assessed overall and specifically in the LGE-positive segments (Table 3, Figure 2), mice receiving transient coronary ligation consistently showed more preserved LV wall thickness than animals enduring persistent ischemia (Figure 2). In contrast, no variations in overall LV thickness or number of LGE-positive segments were detected in sequential CMR studies from the sham group.

### 3.2. 18F-2-Fluoro-2-deoxyglucose PET Imaging

**Figure 3:**
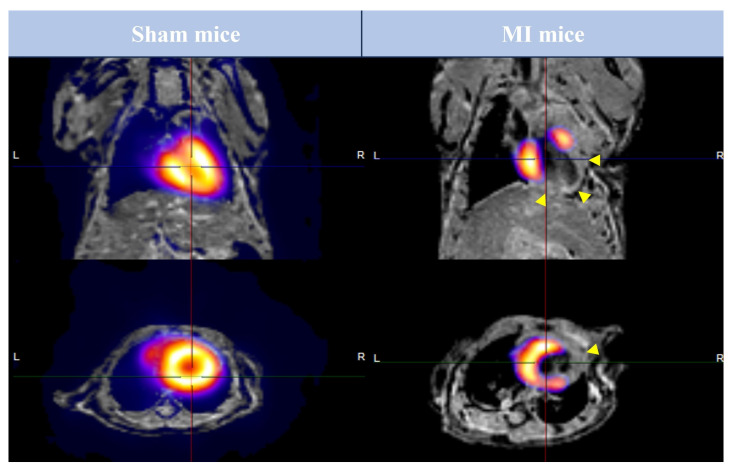
Image fusion of FDG-PET and CMR studies performed in preclinical mice models. Animals from sham (left panel) and MI (right panel) groups were submitted to FDG-PET and CMR imaging 21 days after surgery. Representative pictures in long-axis (upper panel) and short-axis (lower panel) after the fusion of FDG-PET and CMR studies are shown. Arrowheads represent areas with LGE. CMR = Cardiovascular magnetic resonance. FDG = 2-fluor-2-deoxyglucose. LGE = Late gadolinium enhancement. MI = Myocardial infarction. PET = Positron emission tomography.

Following completion of CMR studies, all animals underwent PET imaging tests. Figure 3 illustrates the fusion of CMR/18F-2-fluoro-2-deoxyglucose PET studies performed in sham and MI animals.

A significant reduction in glucose metabolism was noted one day after MI induction compared to baseline, as reflected by an augmented number of segments with low activity and a decay in SUVs. Concretely, SUVs experimented a decrease of 31% (1.3 [1.1–1.6] to 0.9 [0.5–1.2], *p*-value > 0.05) in reperfused and a 32% (1.6 [1.5–2.1] to 1.1 [1.0–1.4], *p*-value < 0.05) in non-reperfused MI mice from 1-day to baseline PET studies. Unlike CMR variables, PET-derived glucose consumption persisted unaltered at the sub-acute and chronic phases after MI induction in both experimental groups (Table 3). In the sham group, glucose consumption was similar in the longitudinal studies. Collectively, significant changes in CMR and PET indices (associated with deteriorating LV systolic function, cardiac architecture, and glucose consumption) were observed within 24 h after MI induction in comparison with the corresponding baseline values or sham animals. In individual examination of the two MI models, mice receiving coronary reperfusion exhibited lower adverse ventricular function, reduced infarct size, and more preserved LV wall thickness at 21-day CMR assessment, whereas no variations were evidenced in PET-derived glucose metabolism.

### 3.3. Prognostic Value of CMR/PET Indices for Histological Infarct Size

**Figure 4:**
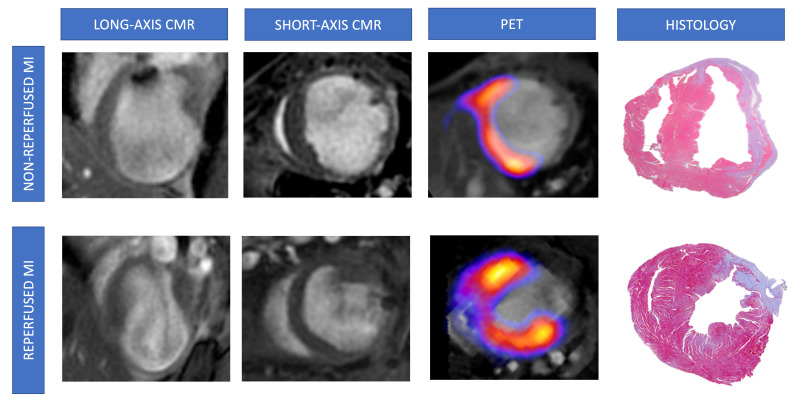
Representative pictures of CMR/FDG-PET studies and histological analysis from mice undergoing non-reperfused and reperfused MI. Animals from non-reperfused (permanent coronary ligation, upper panels) or reperfused (transient coronary ligation, lower panels) MI were submitted to CMR and FDG-PET studies 21 days after surgery and post-mortem histological analysis using Masson’s trichrome staining. Representative images from LGE-CMR (left panel), FDG-PET and CMR fusion (central panel), and microscopic examination (right panel) are shown. CMR = Cardiovascular magnetic resonance. FDG = 2-fluoro-2-deoxyglucose. LGE = Late gadolinium enhancement. MI = Myocardial infarction. PET = Positron emission tomography.

**Figure 5:**
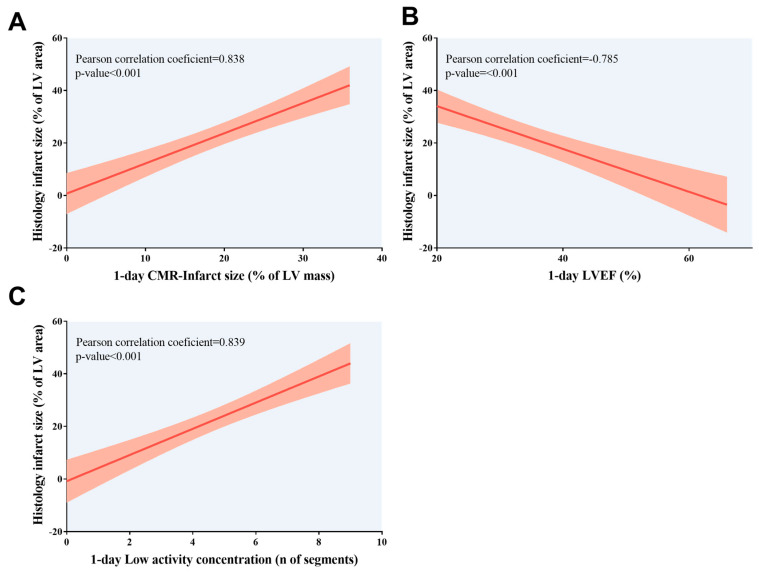
Association of infarct size quantified using histological analysis with 1-day CMR/positron emission tomography indices. Association of higher histology-derived infarct size, expressed as the percentage of LV area, with (**A**) more extensive CMR-derived infarct size, (**B**) depressed LVEF, and (**C**) more segments with low activity concentration according to positron emission tomography examination; both non-invasive cardiac imaging techniques were conducted at 1 day after myocardial infarction induction. CMR = Cardiovascular magnetic resonance. LV = Left ventricle. LVEF = Left ventricular ejection fraction.

Our data showed an association between the extent of histology-derived infarct (expressed as a percentage of LV area) and more extensive CMR-derived infarct size, larger LV volumes, and depressed LVEF (Figure 4). Similarly, a greater number of segments with low activity quantified and a lower SUV in PET conducted one day post-MI induction was correlated with the extent of histology-derived infarct (*p*-value < 0.01 for all comparisons). Similar results were obtained when infarct size was expressed as the number of segments with fibrosis according to microscopic analysis (Table 4, Figure 5). Next, we computed the receiver operating characteristic curve threshold that maximized the sensitivity and specificity of parameters of both imaging techniques (CMR imaging at 1 day: LVEF < 40%, infarct size > 0% and/or ≥1 segment, hypokinesia ≥ 1segment; PET imaging: SUV < 1.44 × 10^−5^, Low concentration of 18F-2-fluoro-2-deoxyglucose ≥ 1 segment). All CMR/PET indices showed a high predictive value for identifying mice submitted to MI according to histology (*p*-value < 0.01 for all comparisons). Table 5 provides the sensitivity and specificity data for each CMR/PET index.

### 3.4. Prognostic Value of 1-Day CMR/PET Indices for LV Remodeling

**Figure 6:**
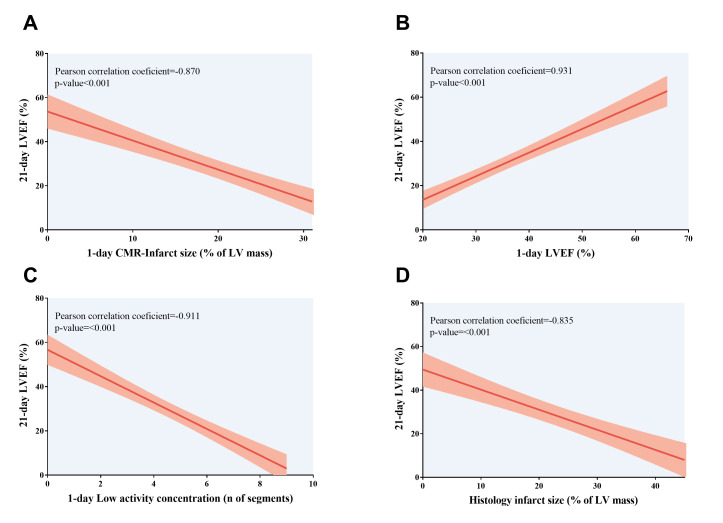
Association of LVEF at 21-day CMR study with 1-day CMR/positron emission tomography indices and histology-derived infarct size. Association of depressed LVEF at chronic (21 days) phase with (**A**) more extensive CMR-derived infarct size, (**B**) depressed LVEF, and (**C**) more segments with low activity concentration according to positron emission tomography examination; both non-invasive cardiac imaging techniques were conducted at 1 day after myocardial infarction induction. (**D**) An indirect correlation was found between 21-day LVEF and histology-derived infarct size, expressed as the percentage of LV area. CMR = Cardiovascular magnetic resonance. LV = Left ventricle. LVEF = Left ventricular ejection fraction.

A greater number of segments with low activity concentration in PET test, higher CMR-derived infarct size, and reduced CMR-LVEF one day after MI induction were associated with worse systolic function at chronic (21-day) phase. Likewise, more depressed systolic function at 21 days was associated with a larger infarct size determined from microscopic analysis (Table 6, Figure 6). As previously performed for histology-derived infarction size, sensitivity and specificity were computed for the receiver operating characteristic curve threshold that maximizes the value of the two parameters. All PET/CMR indices displayed a high-risk classification of LV remodeling, defined as 21-day LVEF, under 40% (*p*-value < 0.01 for all comparisons). Information on the sensitivity and specificity of each CMR index is detailed in Table 5. Altogether, our preclinical models of MI showed that indices derived from non-invasive cardiac imaging techniques performed a few hours after myocardial injury could predict the extent of histology-derived infarct and the occurrence of long-term adverse LV remodeling, as defined by larger LV volumes and depressed LVEF.

## 4. Discussion

This study demonstrates that CMR and 18F-2-fluoro-2-deoxyglucose PET enable early detection of structural, functional, and metabolic alterations in two murine models of MI. Longitudinal evaluation of non-invasive CMR indices are crucial to assess myocardial injury and evaluate the efficacy of novel therapeutic options in preclinical settings.

### 4.1. Importance of Non-Invasive Imaging Techniques in Experimental Models of MI

MI is clinically defined as an abrupt and persistent coronary artery occlusion, most often caused by a thrombus formation following the rupture of an unstable atherosclerotic plaque. Prolonged ischemia and hypoxia ultimately lead to irreversible myocardial damage [1,2]. Despite substantial improvements in survival, MI patients remain at increased risk for recurrent ischemic events, heart failure, and/or arrhythmias [3,4]. Although these risks can be partially mitigated by current standard-of-care therapies, there is a continued need for adjunctive treatments capable of preventing long-term adverse remodeling.

Rodent models are widely used to test novel therapeutic strategies aimed at improving post-MI recovery. However, a persistent translational gap remains between positive clinical results and their limited therapeutic efficacy in patients [5,6,7]. To reduce this discrepancy, robust and reproducible techniques for quantifying infarct size (beyond post-mortem histological staining) must be standardized and validated. In this context, cardiac non-invasive imaging has become a key tool for sequentially characterizing myocardial structure and function during post-MI remodeling in preclinical studies [8,9,10,12,14,15,16,17].

### 4.2. Longitudinal CMR Changes and Comparison Between Permanent and Transient Coronary Ischemia

The introduction of CMR into MI assessment marked a major advance and has established it as the clinical gold standard for non-invasive characterization of structural alterations in ST-segment elevation MI [21,22,23]. However, CMR studies in mice remain technically challenging due to the small cardiac dimensions and rapid heart rates.

In our study, CMR identified early changes in contractile function (reduced LVEF) and myocardial tissue composition (LGE-positive segments) within hours after MI induction in both models. Mice subjected to transient ischemia exhibited larger LGE-defined infarcts and greater regional wall thickening, particularly in LGE-positive segments. Prior echocardiographic studies have shown that reperfusion after 60 min of ischemia results in pronounced reductions in LVEF and progressive LV dilatation, whereas 30 min of ischemia leads to only mild functional decline and no significant remodeling [24]. Ischemia rapidly triggers inflammatory cell recruitment [25,26] and reduces microvascular density within hours after MI [27]. Importantly, reperfusion after prolonged ischemia can induce substantial interstitial edema, potentially leading to overestimation of infarct size on LGE CMR, as reported in both preclinical and clinical studies [19,21,28,29,30].

At chronic time points, marked structural and functional abnormalities persisted in both models. Permanent ischemia resulted in larger LV volumes, more severely depressed LVEF, and thinner infarcted walls. These findings are consistent with echocardiographic studies showing improved systolic function and attenuated remodeling in reperfused versus non-reperfused mice [30]. Although both models are triggered by an identical initial ischemic event, they recapitulate distinct clinical phenotypes and reflect different underlying mechanisms [5,7]. While reperfusion limits infarct size, it may also induce reperfusion injury through oxidative stress, mitochondrial dysfunction, and calcium overload [31,32].

Another variable that might influence the differences between the two MI models is the duration of ischemia in the reperfused MI model, which commonly ranged from 45 to 60 min. Ischemic periods shorter than 45 min tend to produce negligible alterations in myocardial mass, geometry, and function. Conversely, prolonged occlusion exceeding two hours typically results in infarction of nearly the entire area at risk, limiting the ability to assess therapeutic interventions that might exacerbate injury [6,7].

Transient occlusion followed by reperfusion closely models contemporary clinical practice, whereas non-reperfused MI better reflects patients with delayed presentation or failed revascularization who are at higher risk of heart failure. Regardless of model choice, longitudinal CMR provides a robust, non-invasive method for characterizing dynamic infarct evolution and evaluating therapeutic interventions.

### 4.3. Sequential PET Changes in Murine MI Models

18F-2-fluoro-2-deoxyglucose PET analysis demonstrated substantial overlap between LGE-positive segments and regions with reduced glucose uptake, indicating markedly impaired metabolism in infarcted myocardium. Compared with controls, glucose uptake was significantly reduced at 1 day post-MI; however, we did not observe significant temporal changes or differences between the two MI models.

Interpretation of PET findings requires caution, as several experimental variables, including anesthetic protocol, may influence results. Ketamine/xylazine, for example, inhibits translocation of glucose transporters in cardiomyocytes but not in activated leukocytes [12]. Consequently, leukocyte infiltration into infarcted tissue may account for the increased glucose uptake observed under ketamine/xylazine protocols in prior MI studies [12,14]. Regional analysis in ST-elevation MI patients submitted to CMR/PET tests revealed an overlapping between higher 18F-2-fluoro-2-deoxyglucose uptake and LGE [33]. By contrast, our experiments were conducted under isoflurane, which may explain the lower PET signal detected in LGE-positive segments.

Importantly, the number of PET-defined hypometabolic segments at day 1 correlated with histology-based infarct size and long-term LV remodeling. These results align with recent preclinical evidence showing that reduced 18F-2-fluoro-2-deoxyglucose uptake at sub-acute and chronic stages correlates with greater fibrosis [16]. In contrast, in clinical settings, increased 18F-2-fluoro-2-deoxyglucose uptake, reflecting inflammation, has been associated with worse CMR-derived functional outcomes [33].

Overall, our findings highlight the complementary value of CMR and PET for sequential assessment of cardiac function, architecture, and metabolism throughout the post-MI healing process. This combined multimodality approach enhances our ability to evaluate the effects of emerging therapeutics.

### 4.4. Study Limitations

This study focused exclusively on type 1 MI models and did not include animals with type 2 MI, which may exhibit distinct pathophysiological mechanisms and imaging characteristics. This omission may limit generalizability of the findings. Another limitation is the absence of circulating or myocardial leukocyte quantification. Given the central role of the immune response in clearing necrotic tissue and orchestrating scar formation [34], the lack of inflammatory cell data may confound interpretation of PET-derived metabolic indices.

Finally, the small sample size, although not uncommon in mouse studies, may reduce statistical power and limit the generalizability of the findings. Our results should therefore be interpreted with caution and considered preliminary, warranting further studies involving larger experimental groups to validate and extend these observations.

## 5. Conclusions

CMR and 18F-2-fluoro-2-deoxyglucose PET are useful for non-invasive and longitudinal evaluation of variations in the architecture and contractility in preclinical models of MI. Early non-invasive cardiac imaging indices might predict the occurrence of long-term unfavorable LV remodeling, and the magnitude of the resulting infarct size as detected by histology. This approach could ultimately be utilized to monitor and predict the effectiveness of novel maneuvers in the preclinical scenarios.

## Figures and Tables

**Table 1:** Comparison of clinical characteristics and baseline CMR and PET indices between sham mice and both (reperfused and non-reperfused) MI groups.

	Sham	Non-Reperfused MI	Reperfused MI	*p*-Value
Clinical characteristics				
Male (%)	2 (40)	4 (50)	5 (55)	0.855
Age (weeks)	15.8 ± 1.8	16.1 ± 1.9	16.2 ± 1.7	0.918
Weight (g)	23.8 ± 1.7	24.5 ± 2.5	25.3 ± 2.1	0.466
CMR imaging				
LVEF (%)	60.9 ± 9.4	64.5 ± 9.7	59.8 ± 7.3	0.536
LVEDVI (µL)	39.3 ± 5.1	35.8 ± 5.0	35.0 ± 6.1	0.377
LVESVI (µL)	18.3 ± 3.4	14.8 ± 4.4	14.0 ± 8.7	0.482
LV mass (mg)	38.9 ± 3.9	39.3 ± 7.1	40.3 ± 13.8	0.965
IS (% of LV mass)	0.00 ± 0.00	0.00 ± 0.00	0.00 ± 0.00	1.000
IS (*n* of segments)	0.00 ± 0.00	0.00 ± 0.00	0.00 ± 0.00	1.000
Hypokinesia (*n* of segments)	0.00 ± 0.00	0.00 ± 0.00	0.00 ± 0.00	1.000
Global wall thickness(mm)	0.65 ± 0.05	0.67 ± 0.04	0.64 ± 0.04	0.358
18F-FDG PET imaging				
Low activity concentration (*n* of segments)	0.00 ± 0.00	0.00 ± 0.00	0.00 ± 0.00	1.000
SUV of LV (10^5^)	1.5 [0.9–1.5]	1.6 [1.5–2.1]	1.3 [1.1–1.6]	0.194

One control group (*n* = 5) and two mice MI models were formed by permanent coronary ligation (non-reperfused MI, *n* = 8) or transient 45 min (reperfused MI, *n* = 9) coronary occlusion. 18F-FDG = Fluorine-18 2-fluoro-2-deoxyglucose. CMR = Cardiovascular magnetic resonance. IS = Infarct size. LV = Left ventricle. LVEDVI = Left ventricular end-diastolic volume index. LVEF = Left ventricular ejection fraction. LVESVI = Left ventricular end-systolic volume index. MI = Myocardial infarction. PET = Positron emission tomography. SUV = Standard uptake value.

**Table 2:** Inter- and intra-observer variability of histology measurements and PET/CMR characteristics.

	Relative Change	Absolute Change	Intraclass Correlation Coefficient
INTER-OBSERVER VARIABILITY			
Histology			
Infarct size (% of LV area)	7.8 ± 4.3%	2.8 ± 1.9%	0.981
CMR at 1 day			
LVEF (%)	4.4 ± 11.3%	2.6 ± 3.9%	0.983
LVEDVI (µL)	10.0 ± 9.2%	6.4 ± 5.3 µL	0.995
LVESVI (µL)	8.9 ± 10.8%	4.0 ± 3.7 µL	0.998
Infarct size (% of LV mass)	9.4 ± 3.3%	2.7 ± 1.1%	0.986
PET at 1 day			
SUV of LV	11.6 ± 8.9%	0.1 ± 0.1 × 10^−5^	0.959
INTRA-OBSERVER VARIABILITY			
Histology			
Infarct size (% of LV area)	3.6 ± 6.5%	1.7 ± 2.5%	0.989
CMR at 1 day			
LVEF (%)	2 ± 4.6%	0.9 ± 1.9%	0.998
LVEDVI (µL)	3 ± 1.7%	2.4 ± 1.6 µL	0.999
LVESVI (µL)	4.9 ± 5.2%	2.1 ± 1.4 µL	0.999
Infarct size (% of LV mass)	4.1 ± 6.0%	0.9 ± 1.3%	0.997
PET at 1 day			
SUV of LV	3.9 ± 7.2%	0.03 ± 0.05 × 10^−5^	0.982

CMR = Cardiovascular magnetic resonance. LV = Left ventricle. LVEF = Left ventricular ejection fraction. LVEDVI = Left ventricular end-diastolic volume index. LVESVI = Left ventricular end-systolic volume index. PET = Positron emission tomography. SUV = Standard uptake value.

**Table 3:** Dynamic changes in 18F-FDG PET and CMR indices in animals submitted to non-reperfused and reperfused MI.

	Non-Reperfused MI				Reperfused MI			
	Baseline	1 Day	7 Days	21 Days	Baseline	1 Day	7 Days	21 Days
CMR imaging								
LVEF (%)	64.5 ± 9.7	24.5 ± 3.3 **	16.3 ± 4.0 **^/#^	15.7 ± 5.7 **^/##^	59.8 ± 7.3	26.2 ± 9.6***	16.7 ± 5.1 ***^/##^	22.0 ± 5.3 ***^/++^
LVEDVI (µL)	35.8 ± 5.0	62.1 ± 7.8 ***	93.0 ± 20.1 ***^/##^	115.9 ± 31.2 ***^/##/+^	35.0 ± 6.1	70.1 ± 15.1 ***	94.3 ± 26.9 ***^/##^	112.3 ± 32.2 ***^/##/+^
LVESVI (µL)	14.8 ± 4.4	46.8 ± 5.7 ***	78.6 ± 20.1 ***^/##^	99.0 ± 32.0 ***^/##/+^	14.0 ± 8.7	52.9 ± 17.5 ***	79.44 ± 26.0 ***^/##^	88.8 ± 29.7 ***^/##^
LV mass (mg)	39.3 ± 7.1	45.1 ± 4.1	49.3 ± 6.4 *	52.5 ± 7.5 *^/#^	40.3 ± 13.8	52.3 ± 7.0	55.9 ± 11.9	60.2 ± 11.7 *^/#^
IS (% of LV mass)	0.0 ± 0.0	24.7 ± 4.8 ***	27.8 ± 7.5 ***	23.4 ± 5.2 ***^/+^	0.0 ± 0.0	26.3 ± 6.2 ***	22.5 ± 4.5 ***	19.3 ± 2.4 ***^/##^
IS (*n* of segments)	0.0 ± 0.0	5.6 ± 1.7 ***	5.6 ± 1.8 ***	5.6 ± 1.7 ***	0.0 ± 0.0	6.2 ± 0.7 ***	6.2 ± 0.7 ***	6.3 ± 0.7 ***
Hypokinesia (*n* of segments)	0.0 ± 0.0	8.4 ± 2.1 ***	10.6 ± 1.6 ***^/#^	10.9 ± 2.3 ***^/#^	0.0 ± 0.0	10.1 ± 4.2 ***	11.6 ± 2.0 ***	9.9 ± 2.5 ***
Global wall thickness (mm)	0.67 ± 0.04	0.55 ± 0.04 **	0.54 ± 0.05 *	0.51 ± 0.07 *	0.64 ± 0.04	0.62 ± 0.06	0.62 ± 0.08	0.60 ± 0.06
Wall thickness in LGE-positive segments (mm)		0.48 ± 0.06	0.36 ± 0.08 ^#^	0.32 ± 0.11 ^##^		0.56 ± 0.07	0.46 ± 0.10	0.44 ± 0.10
18F-FDG PET imaging								
Low activity concentration (*n* of segments)	0.0 ± 0.0	6.1 ± 0.8 ***	6.3 ± 0.9 ***	5.6 ± 2.5 ***	0.0 ± 0.0	6.0 ± 1.4 ***	5.1 ± 1.5 ***^/##^	5.3 ± 1.4 ***
SUV of LV (10^5^)	1.6 [1.5–2.1]	1.1 [1.0–1.4] *	1.0 [0.8–1.1] *	1.0 [0.9–1.2] *	1.3 [1.1–1.6]	0.9 [0.5–1.2] *	1.0 [0.8–1.2] *	1.0 [0.8–1.5] *

One control group (*n* = 5) and two mice MI models were formed by permanent coronary ligation (non-reperfused MI, *n* = 8) or transient 45 min (reperfused MI, *n* = 9) coronary occlusion. 18F-FDG = fluorine-18 2-fluoro-2-deoxyglucose. CMR = Cardiovascular magnetic resonance. IS = Infarct size. LGE = Late gadolinium enhancement. LV = Left ventricle. LVEF = Left ventricular ejection fraction. LVEDVI = Left ventricular end-diastolic volume index. LVESVI = Left ventricular end-systolic volume index. MI = Myocardial infarction. PET = Positron emission tomography. SUV = Standard uptake value. * *p* < 0.05, ** *p* < 0.01, *** *p* < 0.001 vs. baseline; ^#^ *p* < 0.05, ^##^ *p* < 0.01 vs. 1 day; ^+^ *p* < 0.05, ^++^ *p* < 0.01 vs. 7 days.

**Table 4:** Correlation matrix between histology-derived infarct size and 1-day CMR and PET indices.

	Histology-Infarct Size (% of LV Area)		Histology-Infarct Size (*n* of Segments)	
	Correlation Coefficient	*p*-Value	Correlation Coefficient	*p*-Value
1-day CMR imaging				
LVEF (%)	−0.785	<0.001	−0.707	<0.001
LVEDVI (%)	0.636	<0.001	0.512	0.003
LVESVI (%)	0.729	<0.001	0.615	<0.001
Infarct size (%)	0.838	<0.001	0.843	<0.001
Infarct size (*n* of segments)	0.806	<0.001	0.796	<0.001
Hypokinesia (*n* of segments)	0.783	<0.001	0.720	<0.001
1-day 18F-FDG PET imaging				
Low activity concentration (*n* of segments)	0.839	<0.001	0.860	<0.001
SUV of LV	−0.437	0.016	−0.367	0.046

The association between SUV and histology-derived infarct size was assessed using Spearman’s rank-order correlation, while Pearson correlation coefficient was used in normally distributed variables. 18F-FDG = Fluorine-18 2-fluoro-2-deoxyglucose. CMR = Cardiovascular magnetic resonance. LV = Left ventricle. LVEF = Left ventricular ejection fraction. LVEDVI = Left ventricular end-diastolic volume index. LVESVI = Left ventricular end-systolic volume index. PET = Positron emission tomography. SUV = Standard uptake value.

**Table 5:** AUC of the main CMR/PET characteristics assessed 1 day after myocardial infarction induction to identify mice with infarction and depressed systolic function at follow-up.

	AUC	Sensitivity	Specificity	*p*-Value
Histology-derived infarct size				
1-day CMR-LVEF < 40%	0.94	88.0%	100%	0.003
1-day CMR-Infarct size > 0% of LV mass	1.00	100%	100%	0.002
1-day CMR-Infarct size ≥ 1 segment	1.00	100%	100%	0.001
1-day CMR-Hypokinesia ≥ 1 segment	1.00	100%	100%	0.001
1-day 18F-FDG PET-Low activity concentration ≥ 1 segment	1.00	100%	100%	0.001
1-day PET-SUV of LV < 1.44 × 10^−5^	0.844	93.8%	75%	0.038
21-day CMR-LVEF < 40%				
1-day CMR-LVEF < 40%	0.94	88.0%	100%	0.003
1-day CMR-Infarct size > 0% of LV mass	1.00	100%	100%	<0.001
1-day CMR-Infarct size ≥ 1 segment	1.00	100%	100%	0.001
1-day CMR-Hypokinesia ≥ 1 segment	1.00	100%	100%	<0.001
1-day 18F-FDG PET-Low activity concentration ≥ 1 segment	1.00	100%	100%	<0.001
1-day PET-SUV of LV < 1.44 × 10^−5^	0.64	93.8%	33.3%	0.338

18F-FDG = Fluorine-18 2-fluoro-2-deoxyglucose. AUC = Area under the curve. CMR = Cardiovascular magnetic resonance. LV = Left ventricle. LVEF = Left ventricular ejection fraction. PET = Positron emission tomography. SUV = Standard uptake value.

**Table 6:** Correlation matrix between 21-day systolic function and 1-day CMR, 1-day PET, and histological indices.

	21-Day LVEF (%)		21-Day LVEDVI (%)		21-Dayd LVESVI (%)	
	Correlation Coefficient	*p*-Value	Correlation Coefficient	*p*-Value	Correlation Coefficient	*p*-Value
1-day CMR imaging						
LVEF (%)	0.931	<0.001	−0.775	<0.001	−0.797	<0.001
Infarct size (%)	−0.870	<0.001	0.815	<0.001	0.826	<0.001
Infarct size (*n* of segments)	−0.840	<0.001	0.814	<0.001	0.805	<0.001
Hypokinesia (*n* of segments)	−0.800	<0.001	0.764	<0.001	0.764	<0.001
1-day 18F-FDG PET imaging						
Low activity concentration	−0.911	<0.001	0.840	<0.001	0.850	<0.001
(*n* of segments)						
SUV of LV	0.343	0.109	−0.400	0.059	−0.413	0.051
Histologic analysis						
Infarct size (% of LV area)	−0.835	<0.001	0.805	<0.001	0.822	<0.001
Infarct size (*n* of segments)	−0.818	<0.001	0.706	<0.001	0.743	<0.001

The association between SUV and CMR-derived systolic function at chronic phase was assessed using Spearman’s rank-order correlation, while Pearson correlation coefficient was used in normally distributed variables. 18F-FDG = Fluorine-18 2-fluoro-2-deoxyglucose. CMR = Cardiovascular magnetic resonance. LV = Left ventricle. LVEF = Left ventricular ejection fraction. LVEDVI = Left ventricular end-diastolic volume index. LVESVI = Left ventricular end-systolic volume index. PET = Positron emission tomography. SUV = Standard uptake value.

## Data Availability

The original contributions presented in this study are included in the article. Further inquiries can be directed to the corresponding authors.

## Abbreviations

The following abbreviations are used in this manuscript:

CMR	Cardiovascular magnetic resonance
LGE	Late gadolinium enhancement
LV	Left ventricle
LVEF	Left ventricular ejection fraction
MI	Myocardial infarction
PET	Positron emission tomography
SUV	Standardized uptake value
